# Editorial: Hallmark of cancer: tumor promoting inflammation

**DOI:** 10.3389/fonc.2023.1242407

**Published:** 2023-07-07

**Authors:** Luca Roncati, Carlos R. Figueiredo

**Affiliations:** ^1^ Institute of Pathology, Department of Laboratory Medicine and Anatomical Pathology, University Hospital of Modena – Polyclinic, Modena, Italy; ^2^ Department of Surgery, Medicine, Dentistry and Morphological Sciences with interest in Transplantation, Oncology and Regenerative Medicine, University of Modena and Reggio Emilia, Modena, Italy; ^3^ Medical Immune Oncology Research Group (MIORG), Institute Biomedicine, Faculty of Medicine, University of Turku, Turku, Finland; ^4^ InFLAMES Research Flagship Center, University of Turku, Turku, Finland

**Keywords:** CD1d, pyroptosis, TCGA, IDO1, irAEs, immune checkpoint therapy

## Perspective

Over the past two decades, the Hallmarks of Cancer, introduced by Hanahan and Weinberg, have revolutionized our understanding of cancer’s complex biology, capturing the transformation from healthy to cancerous cells. One of these hallmarks, Tumor Promoting Inflammation, has been identified as a key player in cancer development, providing both protective responses and contributing to tumorigenesis when dysregulated. This Editorial Series focuses on this complex role of inflammation in cancer, aiming to further uncover its mechanisms and implications for resistance tumor growth, resistance to immunotherapies and insights into immune adverse effects, with the ultimate goal of informing novel insights for rational development of safer and more effective treatments.

## Article summary

The articles cover specific aspects of Tumor Promoting Inflammation. Khan et al. conducted a pan-cancer analysis to explore pyroptosis, an inflammatory form of programmed cell death. Their study emphasizes the role of inflammation in tumor development across various cancer types. Pyroptosis, known for its potential as an anti-cancer therapy by triggering anti-cancer immune responses, was the central focus. Using data from The Cancer Genome Atlas (TCGA), the researchers systematically analyzed Pyroptosis-Related Genes (PRGs) expression, genomic aberrations, and clinical significance. Cancers were categorized into pyroptosis-low and pyroptosis-high groups based on a Gene Set Variation Analysis (GSVA) score. Immunohistochemistry assessed PRGs expression in specific tumor types. The findings revealed differential PRGs expression across cancers, impacting prognosis. Genomic and epigenetic abnormalities, including Single-Nucleotide Variants (SNVs), Copy Number Variation (CNV), and DNA methylation, influenced these expression variations. Methylation of PRGs predicted improved survival in certain cancers, like Lower Grade Glioma (LGG), Uveal Melanoma (UVM), and Kidney Renal Clear Cell Carcinoma (KIRC), while upregulation posed higher risks. Pyroptosis levels distinguished tumor from normal samples in multiple cancer types and correlated with cancer stage and prognosis. Higher pyroptosis levels were associated with worse outcomes in overall survival, progression-free survival, and disease-specific survival. Elevated pyroptosis levels indicated a more immune-active tumor microenvironment with increased CD8+ T cells and other subtypes. Oncogenic pathways were enriched in pyroptosis-high subgroups across cancers.

Taking a unique angle, Wang et al. theorize about the epigenetic control of Cluster of Differentiation 1D (*CD1D*) expression in poorly immunogenic melanomas as a mechanism of Immune Checkpoint Therapy (ICT) resistance, providing strong preliminary clinical evidence for reverse translational research. The authors probe the connection between β2-microglobulin (β2M) deficiency - a factor known to inhibit antigen presentation to T cells - and ICT resistance. Using the Search Tool for the Retrieval of Interacting Genes/Proteins (STRING) database, they single out immune biomarkers interacting with β2M, followed by analysis of these markers’ transcriptomic expressions in the Genomic Data Commons (GDC) - TCGA - Skin Cutaneous Melanoma (SKCM) dataset and in multiple ICT-treated metastatic melanoma cohorts. Further investigation into the epigenetic control of these biomarkers is done using the Illumina Human Methylation 450 dataset. The findings suggest a link between β2M and CD1d, CD1b, and Fc fragment of IgG Receptor and Transporter (FCGRT) proteins. This association, however, diverges when β2M expression is lost in melanoma patients. Lower *CD1D* expression, commonly found in patients with worse survival rates and resistance to anti-PD1 therapies, is highlighted. Enhanced β2M and CD1D presence in tumor cells and dendritic cells is noted in patients responding positively to anti-PD1 treatments, along with a higher frequency of natural killer T cell signatures in the tumor environment. Lastly, the authors propose that methylation reactions affecting *B2M* and Spi-1 Proto-Oncogene (*SPI1*) gene expression (which modulates *CD1D* expression) might have crucial implications on β2M and CD1d functions. These insights offer a promising direction towards developing rational combinatory therapies for metastatic melanoma patients with resistance to anti-PD1 immunotherapies.

Muller et al.’s comprehensive review scrutinizes the role of Indoleamine 2,3-Dioxygenase 1 (IDO1), a key player in immune evasion, in promoting tumor neovascularization and its crosstalk with the inflammatory microenvironment. The authors emphasize that chronic Tumor Promoting Inflammation forms a microenvironment conducive to cancer growth, and that distinguishing the factors contributing to such inflammation is vital. This review article highlights the indispensable role of Tumor Promoting Inflammation in neoplasia and metastatic progression. Among the key contributors is IDO1, a tryptophan catabolizing enzyme implicated in both immunometabolism and inflamometabolism. IDO1 aids tumor immunity evasion by fostering immune tolerance to tumor antigens. It also stimulates tumor neovascularization by influencing local innate immunity, a role mediated by a myeloid cell group named IDO1-Dependent Vascularizing Cells (IDVCs). These cells, initially identified in metastatic lesions, might influence pathological neovascularization in various diseases. IFNγ-induced IDO1 expression in IDVCs triggers the production of the pro-angiogenic cytokine IL-6, thereby facilitating vascular access for the tumor. This function of IDO1 complements its contribution to other cancer hallmarks, possibly originating from its role in physiological processes like wound healing and pregnancy. Comprehending how IDO1’s role varies across different tumors will be essential in devising effective IDO1-targeted therapies.

Lastly, the case report by Marie et al. navigates on two Non-Small Cell Lung Cancer (NSCLC) patients treated with nivolumab (anti-PD1), both of whom developed Checkpoint Inhibitor-induced Thyroid Dysfunction (CITD) followed by further immune-related Adverse Events (irAEs), specifically pneumonitis and intestinal perforation. Notably, the onset of CITD was associated with an increase in peripheral CD8+ T cells in these patients. However, traditional inflammatory markers, such as C-Reactive Protein (CRP) and the Neutrophil/Lymphocyte Ratio (NLR), did not consistently elevate during CITD onset, but showed a significant increase during the onset of pneumonitis and intestinal perforation irAEs. Marie et al.’s findings imply that while CRP levels and NLR may not be indicative of CITD, T cell expansion might be indicative of immunotherapy-induced thyroiditis.

As Editors of this Research Topic, we hope that the selected articles will provide a platform for novel hypotheses and future research directions on Tumor Promoting Inflammation in this Editorial Series, one of the core Hallmarks of Cancer. These investigations are revealing intricate biomarkers, molecular and cellular processes at play in the tumor microenvironment, underlining the importance of inflammation in the promotion, evasion of cancer growth and therapeutic development. We hope that this Research Topic will serve as a stimulus for further research and innovation in the pursuit of more effective, personalized, and safe treatments.

## Author contributions

LR revised critically the manuscript for intellectual content; CF drafted the manuscript and revised it critically for intellectual content. Both the authors agree with the final version of the manuscript.

